# Immune Reaction and Survivability of *Salmonella* Typhimurium and *Salmonella* Infantis after Infection of Primary Avian Macrophages

**DOI:** 10.1371/journal.pone.0122540

**Published:** 2015-03-26

**Authors:** Maria Braukmann, Ulrich Methner, Angela Berndt

**Affiliations:** 1 Institute of Molecular Pathogenesis, ‘Friedrich-Loeffler-Institut’ (Federal Research Institute for Animal Health), Jena, Germany; 2 Institute of Bacterial Infections and Zoonoses, ‘Friedrich-Loeffler-Institut’ (Federal Research Institute for Animal Health), Jena, Germany; Ross University School of Veterinary Medicine, SAINT KITTS AND NEVIS

## Abstract

*Salmonella* serovars are differentially able to infect chickens. The underlying causes are not yet fully understood. Aim of the present study was to elucidate the importance of *Salmonella* Pathogenicity Island 1 and 2 (SPI-1 and -2) for the virulence of two non-host-specific, but *in-vivo* differently invasive, *Salmonella* serovars in conjunction with the immune reaction of the host. Primary avian splenic macrophages were inoculated with *Salmonella enterica* sub-species *enterica* serovar (S.) Typhimurium and *S*. Infantis. The number and viability of intracellular bacteria and transcription of SPI-1 and -2 genes by the pathogens, as well as transcription of immune-related proteins, surface antigen expression and nitric oxide production by the macrophages, were compared at different times post inoculation. After infection, both of the *Salmonella* serovars were found inside the primary macrophages. Invasion-associated SPI-1 genes were significantly higher transcribed in *S*. Infantis- than *S*. Typhimurium-infected macrophages. The macrophages counteracted the *S*. Infantis and *S*. Typhimurium infection with elevated mRNA expression of inducible nitric oxide synthase (iNOS), interleukin (IL)-12, IL-18 and lipopolysaccharide-induced tumor necrosis factor alpha factor (LITAF) as well as with an increased synthesis of nitric oxide. Despite these host cell attacks, *S*. Typhimurium was better able than *S*. Infantis to survive within the macrophages and transcribed higher rates of the SPI-2 genes *spiC*, *ssaV*, *sifA*, and *sseA*. The results showed similar immune reactions of primary macrophages after infection with both of the *Salmonella* strains. The more rapid and stronger transcription of SPI-2-related genes by intracellular *S*. Typhimurium compared to *S*. Infantis might be responsible for its better survival in avian primary macrophages.

## Introduction

The genus *Salmonella* is characterized by a high serological diversity and contains more than 2600 serovars [1]. In chickens, as in mammals, *Salmonella* organisms colonize the intestinal tract and penetrate the mucosal epithelium in the distal ileum and cecum, which is typically followed by the development of gastroenteritis. After passing the epithelial cells of the gut wall, the *Salmonella* organisms encounter a set of resident macrophages lining the inner side of the mucosal epithelium. These macrophages are the first immune defender cells the pathogens have to deal with. *In-vitro* studies have shown on the one hand that avian macrophages possess very efficient bactericidal functions against *Salmonella* [2]. On the other hand, *Salmonella* is equipped to survive within cultured chicken macrophages [3–5].

Former studies of our group demonstrated different abilities of the *Salmonella enterica* sub-species *enterica* serovar (*S*.) serovars Typhimurium and Infantis to invade lower regions of the cecal lamina propria *in-vivo*. As shown, *S*. Infantis entered solely epithelial cells and regions located directly below the epithelium (sub-epithelial region), whereas *S*. Typhimurium was able to spread over the whole lamina propria and reached also the deeper location of the cecal mucosa [6]. How far intestinal macrophages control the invasiveness and dissemination of different non-host-specific *Salmonella* serovars and, thus, the final outcome of the infection *in-vivo* is unknown. It has been postulated that the ability to persist and multiply within chicken macrophages is essential for *Salmonella* pathogenesis and the establishment of a systemic infection [7]. To stop *Salmonella* expansion and promote a sufficient host’s defense, activated macrophages can produce a set of bactericidal substances and/or immune mediators and present parts of the pathogen on their surface. For chickens it has been shown that *Salmonella*-stimulated phagocytes of the HD11 cell line or primary macrophages enhance their production of the immune mediator and bactericidal substance nitric oxide (NO) and increase the transcription levels of different cytokines and chemokines [8–11]. However, a comparison of the triggered immune response of primary avian macrophages after stimulation with divergent non-host-specific *Salmonella* serovars, in connection with virulence gene expression by these pathogens, has never been done before.

To enter the host and initiate enteropathogenesis, *Salmonella* organisms are equipped with a set of important factors genetically determined in two type III secretion systems (TTSS-1 and TTSS-2) encoded on *Salmonella* Pathogenicity Islands (SPI), especially SPI-1 and SPI-2. Of the 21 SPIs known to date, the SPI-1 and SPI-2 are the most studied [12]. The SPI-1 is essential for invasion and initiation of enteropathogenesis [13]. In contrast, the SPI-2-encoded genes are needed for bacterial metabolism and replication within the host cell [14,15]. Previous studies have already shown that SPI-1 is essential for *Salmonella* colonization in gut [16] whereas both SPI-1 and SPI-2 are required for colonization in spleen and liver [16,17].

The present study was undertaken to unravel the importance of SPI-1 and -2 for the virulence capacity of two *Salmonella* serovars, which were formerly found to be differentially able to invade lower regions of the cecal mucosa and to elicit immune reactions in tissue of very young chicks [6], in conjunction with the immunological response of the host cells. Following *in-vitro* infection of primary avian macrophages with *S*. Typhimurium or *S*. Infantis, we determined the number, intracellular survivability and virulence-associated gene expression of the *Salmonella* strains and simultaneously analyzed the antigen expression, transcription of cytokines and production of nitric oxide by the macrophages.

## Materials and Methods

### Chickens

Specific-pathogen-free White Leghorn chickens were hatched from eggs (Charles River GmbH, Extertal, Germany) and housed in floor management at the facilities of ‘Friedrich-Loeffler-Institut’ (Jena, Germany). Animal housing was in accordance with the guidelines for animal welfare set forth by the European Community. The chickens were reared and kept under standardized conditions (room climate: 18–20°C, rel. humidity: 50–60%) throughout the entire study. Antibiotic-free commercial feed in powder form and drinking water were both available *ad libitum*.

This study was carried out in strict accordance with European and National Law for the Care and Use of Animals as well as the German Animal Welfare Act. According to these, no specific approval is needed for sacrificing animals and the collection of organs or tissues for scientific use. The study was conducted after ethical approval and under supervision of the authorized institutional Agent for Animal Protection of the ‘Friedrich-Loeffler-Institut’, Germany.

During the entire study, every effort was made to minimize animal suffering. There were no manipulations or treatments of the animals. The sacrificing of the animals was carried out in strict accordance with the German Animal Welfare Act (BGBl I No. 36, 12. July 2013) and the Commission Regulation implementing Directive 2010/63/EU of the European Parliament and the Council regarding the protection of animals used for experimental and other scientific purposes (Germany, BGB1 No. 47, 12. August 2013). Sacrificing was accomplished by authorized personal staff of the ‘Friedrich-Loeffler-Institut’. All 39 chickens were sacrificed by percussive blow to the head (severe concussion) and immediate bleeding.

### Bacterial strains

*Salmonella enterica* subsp. *Enterica* (*S*.) serovar Typhimurium 9098 and serovar Infantis 1326 [6,18] were used to inoculate avian macrophages. Both *Salmonella* strains were stored in the Microbank system (PRO-LAB Diagnostics, Ontario, Canada) at -20°C. The bacterial suspensions for infection were cultivated by shaking in nutrient broth (SIFIN, Berlin, Germany) for 18 h at 37°C. Infection doses were estimated by measuring extinction at 600 nm against a calibration graph determined for each strain and subsequent plate counting on nutrient agar (SIFIN).

### Isolation and culture conditions of avian splenic macrophages

The primary macrophages from the spleens of 39 chickens aged more than three months were separated by the use of an adapted panning technique as follows. After sacrificing and exsanguinating the donor, spleens were aseptically removed and immediately placed in 2 ml phosphate-buffered saline (PBS) containing 1 mg/ml gentamicin (Life technologies, Darmstadt, Germany). Organs were gently crushed to isolate immune cells. The remaining pieces of connective tissue were discarded from the cell suspension. Afterwards, mononuclear cells were separated by density gradient centrifugation (Histopaque-1077, Sigma-Aldrich, Munich, Germany). A centrifuge tube containing 2 ml Histopaque-1077 solution was carefully loaded with 2 ml of the cell suspension and centrifuged at 250 x g for 40 minutes. Subsequently, splenic mononuclear cells were collected from the interface and washed in 5 ml RPMI-1640 medium added with L-glutamine (PAN-Biotech, Aidenbach, Germany) and containing 5% fetal calf serum (FCS, PAN-Biotech), 5% heat-inactivated chicken serum (chS, Sigma-Aldrich) and 50 μg/ml gentamicin at 300 x g for 15 minutes. Supernatant was removed and the cell pellet re-suspended in 10 ml RPMI-1640 medium (5% FCS, 5% chS, 50 μg/ml gentamicin). Finally, the cell concentration was determined using a 0.0025 mm^2^ Neubauer counter chamber (Paul Marienfeld, Lauda-Königshofen, Germany). Cells were seeded in flasks T25 (Corning Life Sciences, Schiphol-Rijk, The Netherlands) at a density of 9 x 10^6^ isolated splenic cells per ml. Incubations were performed at 41°C in the presence of 5% CO_2_. After 4 h of adherence, the cells were washed twice with PBS (41°C) to remove non-adherent cells, and fresh RPMI-1640 medium (5% FCS, 5% chS, 50 μg/ml gentamicin) was added. The culture was kept under the same conditions with changing the medium every three days.

### Experimental infection

On day 8 of primary culture, macrophages had formed a nearly confluent monolayer. For *Salmonella* infection, medium and non-adherent cells were removed and the remaining cells washed. One cultured flask was used to determine the exact number of macrophages per ml medium. For that, the adherent macrophages were removed by the use of Accutase (30 min, 37°C, 5% CO_2_; Sigma-Aldrich) and counted. After that, the bacterial strains *S*. Typhimurium or *S*. Infantis were added to the cell cultures at a multiplicity of infection (MOI) of 1, 10 or 100. At 4 h after co-cultivation, cells were washed twice with PBS (41°C) to remove unbound bacteria and incubated with fresh medium containing 100 μg/ml gentamicin. Controls were treated as infected cells but without bacteria inoculation. Samples were taken at 1 h, 2 h, 4 h, 24 h and 48 h after *Salmonella* infection.

### Flow cytometry

For characterization of the avian splenic macrophages and analysis of changes in macrophage antigen-expression intensities, apoptosis and cell destruction in consequence of the *Salmonella* infection, avian macrophages were harvested using Accutase (Sigma-Aldrich). Antigen expression was detected by staining with fluorescein isothiocyanate (FITC)-labeled mouse monoclonal antibodies against CD44 (clone AV6), αβ(Vß_2_) T cells (clone TCR2), γδ T cells (clone TCR1), B lymphocytes (BU1; clone AV20) and the avian major histocompatibility complex (MHC)-I and -II (clone F21-2 and 2G11, respectively; all from Southern Biotechnology Associates, Eching, Germany). To prove the macrophage phenotype, the avian macrophage-specific mouse monoclonal antibody KUL01-FITC (Southern Biotechnology Associates) [19] was applied. Optimal antibody concentrations and dilutions were tested. 3 x 10^5^ primary macrophages were incubated with the appropriate antibody in the dark for 30 min. To exclude and determine the number of dead cells, samples were additionally stained with a propidium iodide solution (end concentration: 20 pg/100 μl cell suspension) for 2 min. Aliquots of 10,000 cells per sample were analyzed using a FACSCalibur flow cytometry system (Becton Dickinson, Heidelberg, Germany) equipped with a 15 mW, 488 nm argon ion laser.

For detection of apoptotic cells, an Annexin V Fluos staining (Boehringer, Mannheim, Germany) was performed. Prior flow cytometry, macrophages (1 x 10^6^) were washed in 3 ml Hepes-buffer (10 mM Hepes/NaOH, 140 mM NaCl, 5 mM CaCl_2_) at 250 x g for 10 min, resuspended in 100 μl Annexin V Fluos staining solution (1 ml Hepes-buffer, 20 μl Annexin V Fluos, 1 μg propidium iodide) and incubated for 15 min at 4°C.

To determine the percentage of *Salmonella*-containing macrophages, the BD Cytofix/Cytoperm Fixation/Permeabilization kit (Becton Dickinson) was used according to the manufacturer’s instructions. After permeabilizing of macrophages, the intracellular as well as surface-attached bacteria were stained with an anti-*Salmonella* LPS antibody (ViroStat, Portland, USA) in combination with a FITC-labeled secondary goat anti-mouse immunoglobulin (Dako, Hamburg, Germany) for 20 min. Macrophage surface-attached bacteria were stained by use of the anti-*Salmonella* antibody and the secondary FITC-labeled immunoglobulin without previous cell permeabilization. The percentage of macrophages with intracellular bacteria was calculated by subtraction of the percentage of positive macrophages without permeabilization from the percentage of positive macrophages after permeabilization.

Additionally, the increase of the SSC geometric mean was determined after infection of the cultured macrophages with *S*. Infantis or *S*. Typhimurium. This parameter additionally reflects the magnitude of *Salmonella* invasion into or *Salmonella* uptake by the macrophages. However, a differentiation between surface-attached and intracellular bacteria is impossible with this method.

### Bacteriology

To estimate the number of living intracellular *Salmonella* organisms, 1000 macrophages (adjusted after counting of the detached culture cells using a Neubauer counter chamber) in 100 μl PBS supplemented with 0.01% Triton X-100 (Sigma-Aldrich) were lysed for 20 min. After plating the suspension in 10-fold dilutions on XLD agar (Carl Roth, Karlsruhe, Germany) and incubation at 37°C for 15–24 h, the colonies were counted and numbers of intracellular living bacteria per macrophage calculated by dividing the number of bacterial colonies by the number of plated macrophages. Results are shown as mean values of all dilutions.

### Phase contrast

Phase contrast microscopy was used to characterize the development and growth of the primary avian splenic macrophages and to control the cell culture. For that, an inverse microscope (Leica, Bensheim, Germany) was used. Microscopic analysis was performed between 1 and 7 days after seeding the cells into the flasks.

### Quantitative real-time reverse transcription (RT)-PCR

Samples collected from control and infected primary cell cultures were lysed using RLT-buffer (Qiagen, Hilden, Germany) containing 0.14 M 2-mercaptoethanol (Merck, Darmstadt, Germany) and stored at -80°C. Total RNA was extracted using the RNeasy Mini Kit (Qiagen) following the manufacturer’s instructions. Residual DNA was digested using the RNase-free DNase set (Qiagen). Extracted RNA was eluted in 50 μl RNase-free water (Qiagen) and stored at -80°C. Quantity and quality of mRNA were checked by spectral analysis (BioPhotometer, Eppendorf, Hamburg, Germany). Only samples that fulfilled purity criteria of A_260_/A_280_ ratio of 1.8 to 2.2 and of A_260_/A_230_ ratio of > 2 were included in the study.

The avian-specific primers for IL-8 [20], IL-10, IL-12β (p40), IL-15 [21], IL-18, LITAF, iNOS [6], and Toll-like receptors (TLR3, TLR4) [22] were used. Primer design has already been described [6].

Primers for the *Salmonella* virulence genes *hilA*, *hilC*, *sifA*, *spiC*, *ssaV*, *sseA*, *sseG*, *invF*, *sipA*, and *ssaB* were designed using Primer Express 2.0 software (Applied Biosystems, Darmstadt, Germany) on the basis of genome sequence of *S*. Enteritidis and *S*. Typhimurium available from the Wellcome Trust Sanger Institute (http://www.sanger.ac.uk/Projects/Salmonella). The primer sequences and annealing temperatures are listed in Table 1 [23]. All *Salmonella*-specific primers were verified by BLAST analysis against the chicken genome to rule out cross-reactivity with host genes. Each primer pair was tested by serial dilution of template RNA to ensure optimized amplification and comparable efficiencies of the RT-PCR. For *S*. Infantis and *S*. Typhimurium, the gene expression of the virulence genes and the suitability of the primers were additionally tested prior to starting the experiments.

**Table 1:** *Salmonella* primer sequences for quantitative real-time RT-PCR.

*Salmonella* cDNA target		Sequence 5’ → 3’	Annealing temperature	Accession no.^*a*^
**16S**	Forward:	ACTTGGAGGTTGTGCCCTTGAG	57°C	NC_003197
	Reverse:	GCCCCCGTCAATTCATTTGA		
***invF***	Forward:	CCATTTTCGTCGTTTGTGCA	56°C	U08280
	Reverse:	AATTGGGTGATGTTCTCGTGG		
***hilA***	Forward:	ATCGCAGTATGCGCCCTTT	57°C	AY712754
	Reverse:	ATCCTTCCCATCGGGTATCAT		
***hilC***	Forward:	AGCGATCTCACCCGCAAAT	57°C	NC_003197
	Reverse:	AAAACTCACCTCTTCAGCGGC		
***sifA***	Forward:	GAAAGCGCAAGAAAAGGCAA	57°C	NC_003197
	Reverse:	GGTTTTAAACCACACAGCGCC		
***sipA***	Forward:	AGACCGAGATCAAAACGCAGG	57°C	U40013
	Reverse:	TCAGCGCGGGAAAATCTTC		
***spiC***	Forward:	GAAGGTAATAGCCGATCCTGGA	56°C	NC_003197
	Reverse:	TCACATAGGCAAGACAAGGCTT		
***ssaB***	Forward:	CTGAGGAGGGATTCATGCTGG	55°C	AE008761
	Reverse:	AGGATCGGCTATTACCTTCGG		
***ssaV***	Forward:	TATTGATAGGCGCGGACGCTA	57°C	Y09357
	Reverse:	CGCCTTATGGGCCATGTCTTT		
***sseA***	Forward:	CCGGGCTAAGGTGAGTCAAC	56°C	NC_003197
	Reverse:	GGCAACGCCTTGTGGAAAT		
***sseG***	Forward:	GATTTTATGCTGTTCCGCTGG	56°C	NC_003197
	Reverse:	ACATCCGCGATGGCAATAG		

^a^ NCBI accession number

To determine mRNA expression rates, the QuantiTect SYBR Green real-time one-step RT-PCR kit (Qiagen) was used as described previously [6,23]. The Mx3000P real-time PCR thermocycler (Agilent Technologies, CA, USA) was used with the following temperature/time profile: 50°C/30 min and 95°C/15 min, 45 cycles of 94°C/15 s, and the optimized annealing temperature (Table 1) for 30 s, followed by 72°C for 30 sec. To check the amplified products for specificity, subsequent melting curve analysis was conducted as follows: 95°C for 1 min, 55°C for 30 sec, and 95°C for 30 sec. The threshold method was used for relative quantification of mRNA levels [24]. Normalization of target genes was performed using glycerinaldehyde-3-phosphat (GAPDH) as an endogenous standard for chicken genes and specific 16S rRNA for *Salmonella* genes. For every primer, no-template controls were performed. For every *Salmonella* virulence gene sample, additional RT-PCR controls were implemented by use of master mixes without the reverse transcriptase. Results were expressed as fold changes (2^-ΔΔ*CT*^ method) [24] from the amount of transcripts detected after mock infection (MOI 0) or bacteria before infection.

### Nitrite assay

The level of nitrite as a reflection of nitric oxide (NO) production was measured in culture supernatants using the modified Griess reagent (Sigma-Aldrich). In brief, aliquots of 50 μl freeze-thawed samples were mixed with an equal volume of 1 x Griess reagent and incubated for 15 min at room temperature. The nitric oxide concentration was estimated by measuring the absorbance at 540 nm (ELISA reader, Rainbow, Tecan, Crailsheim, Germany) against a calibration curve using sodium nitrite (Sigma-Aldrich) as a standard. The absorbance of medium alone was used as blank.

### Statistical analysis

The normally distributed data of quantitative real-time RT-PCR, flow cytometry and nitrite assay were statistically evaluated using the Student’s *t* test. This test was also used for comparison of two independent samples to evaluate significant differences between infected cells (*S*. Typhimurium and *S*. Infantis) and controls. Statistical analysis of data from bacteriology was conducted using the unpaired Mann-Whitney U test, because data were not normally distributed (Kolmogorov Smirnov adaptation test, SPSS 19.0). Differences were considered significant for *P* values of ≤ 0.05 and tending to be significant for *P* values of ≤ 0.1.

## Results

### Phenotype of cultured avian macrophages

**Fig 1:**
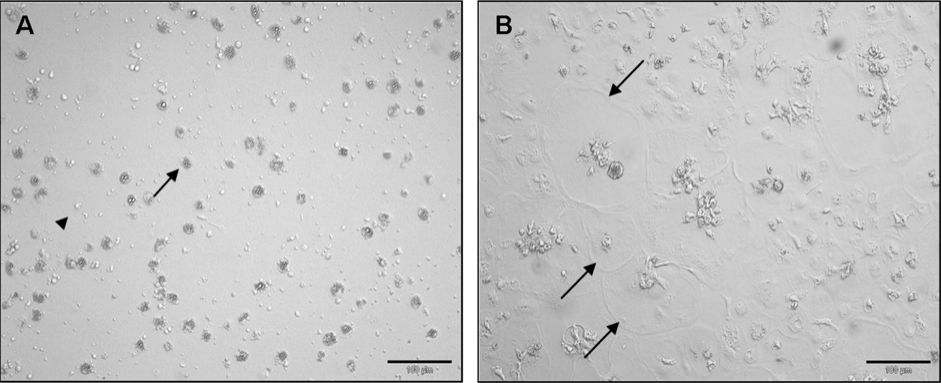
Phase contrast micrographs of primary avian macrophages. **A)** Heterogeneous cell population at day 1 after isolation. **B)** Day 7 of primary cell culture, monocytes differentiated into flattened circular macrophages with extensive cytoplasmic veil. arrowhead: lymphocyte, arrow: primary macrophage, bar = 100 μm.

Using phase contrast microscopy, monocytes matured to round cells with a diameter between 30–150 μm within 7 days of culture (Fig. 1). Similar to human macrophages, the differentiation of avian macrophages was associated with a reduction in the nucleo-cytoplasmic ratio due to an increase in cytoplasmic volume [25].

**Fig 2:**
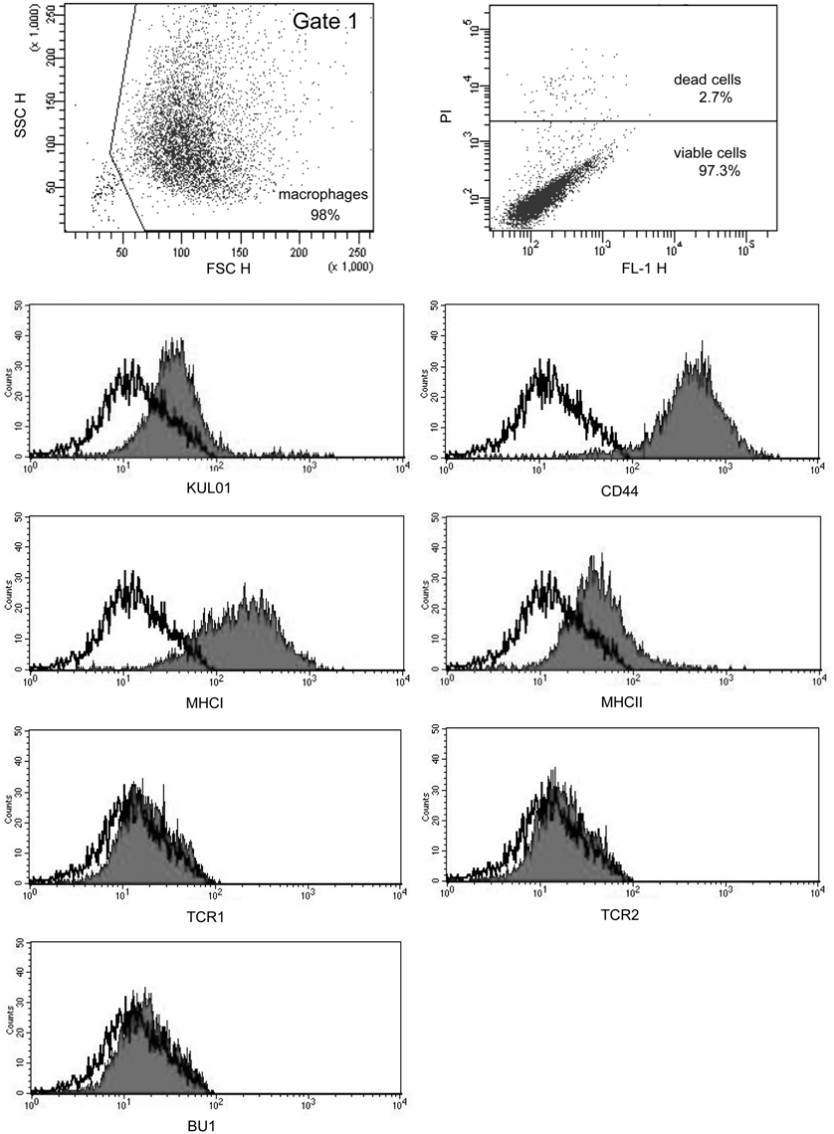
Flow cytometric characterisation of primary avian macrophages. FSC-SSC dot plot diagram shows primary avian macrophages as large and granular cells at day 7 of culture. Dead cells were stained using propidium iodide and represented approximately 2.7% of avian macrophages. Representative histograms indicate expression of cell-surface molecules on primary avian macrophages (gate 1) cultured for 7 days. Multigraph overlay from flow cytometry analysis are shown. The black line represents the isotype control, the gray peak shows cells labeled with the appropriate monoclonal antibody. Cultured macrophages were positively stained with the antibodies against chicken monocytes/macrophages—KUL01, CD44, MHC class I and II. Cultured macrophages were negatively stained for B cells (BU1), γδ T cells (TCR1) and αβ T cells (TCR2). n = 8

Flow cytometry was used to characterize the surface antigen expression of the primary avian macrophages at day 7 of culture (Fig. 2). Within the FSC/SSC dot plot diagram, macrophages (gate 1, approximately 98.0%) were seen as large, granular and living (approximately 97.3%) cells. The macrophages in gate 1 were positively stained for the monocyte/macrophage marker KUL01 [19] and expressed the antigens CD44, MHC class I and II (Fig. 2).

### Calculation of numbers of *Salmonella*-containing macrophages

**Fig 3:**
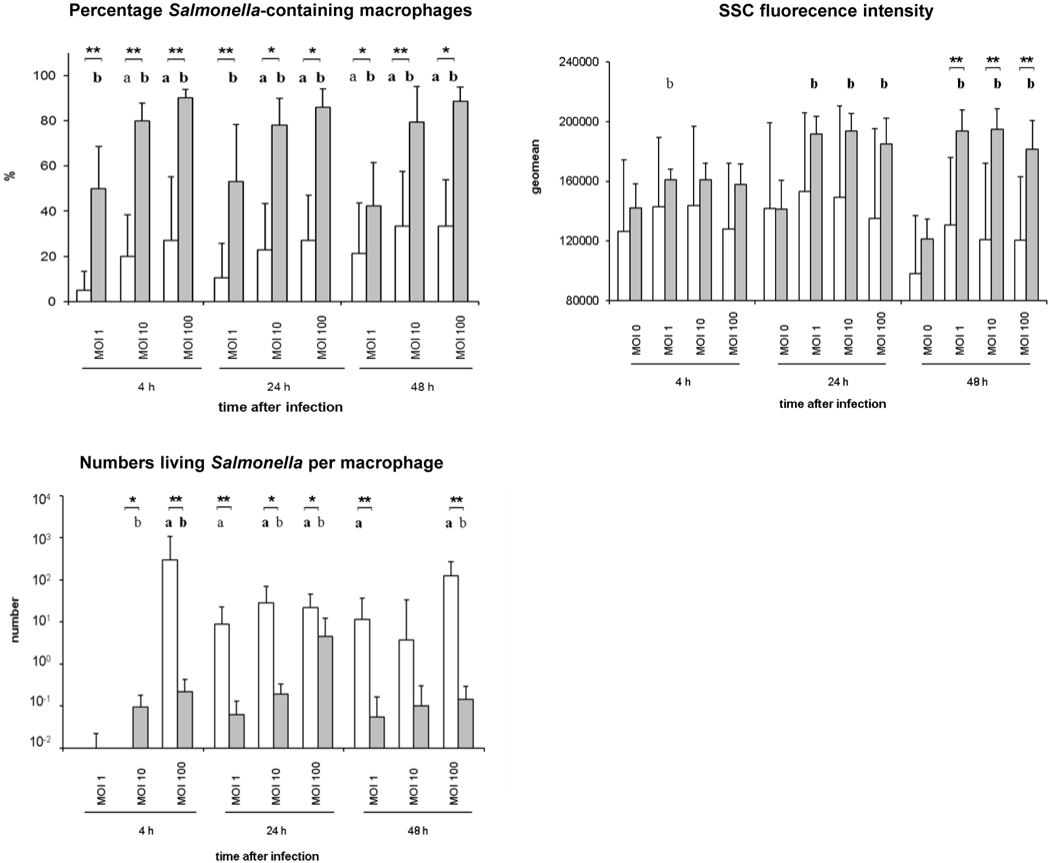
Quantification of *Salmonella*-containing macrophages (flow cytometry) and numbers of intracellular bacteria (microbiology). The percentage of avian macrophages containing intracellular *Salmonella* was analyzed by flow cytometry after staining with an anti-*Salmonella* LPS antibody. Additionally, the SSC fluorescence intensity, which correlates with the *Salmonella* invasion/uptake, was determined after *Salmonella* infection of the primary macrophages. Numbers of viable intracellular *Salmonella* per avian macrophages were determined using bacteriology. White columns—macrophages infected with *S*. Typhimurium (n = 8), Grey columns—macrophages infected with *S*. Infantis (n = 6) ** *P* ≤ 0.05 or * *P* ≤ 0.1 *S*. Typhimurium vs. *S*. Infantis; **a**) *P* ≤ 0.05 or a) *P* ≤ 0.1 *S*. Typhimurium vs. control; **b**) *P* ≤ 0.05 or b) *P* ≤ 0.1 *S*. Infantis vs. control.

The percentage of macrophages containing intracellular *Salmonella* was determined using flow cytometry (Fig. 3). By staining of intracellular *Salmonella* organisms, significantly higher percentages of *Salmonella*-containing macrophages were calculated after *S*. Infantis inoculation compared to *S*. Typhimurium. Whereas up to 90% of macrophages were *S*. Infantis-positive (MOI 100), the infection with *S*. Typhimurium (MOI 1, 10 and 100) resulted in less than 40% infected macrophages at 4, 24 and 48 hpi (Fig. 3).

To corroborate these data, the intensity (geometric mean) of the SSC light of the macrophages after infection was examined additionally (Fig. 3). Although standard deviations were relatively high, the results showed an increase of the geometric mean of the SSC fluorescence intensity in *S*. Infantis- and *S*. Typhimurium-infected macrophages compared to non-infected cells. Significant differences between the strains were only seen at 48 hpi. Similar to the results of the *Salmonella* fluorescence staining, highest values were found after *S*. Infantis infection of the avian primary splenic macrophages.

### Survivability of *Salmonella* in primary macrophages

The microbiological agar plating method was used to determine the number of viable intracellular *Salmonella* per macrophage. After infection of the macrophage culture, significantly higher numbers of living intracellular *S*. Typhimurium than *S*. Infantis were found per macrophage (Fig. 3). Whereas up to 125 *S*. Typhimurium organisms were detected within a single avian macrophage at 48 hpi (MOI 100), the infection with *S*. Infantis resulted in a maximum of 4 live bacteria per macrophage at 24 hpi (MOI 100).

### Macrophage viability and apoptosis after *Salmonella* infection

**Fig 4:**
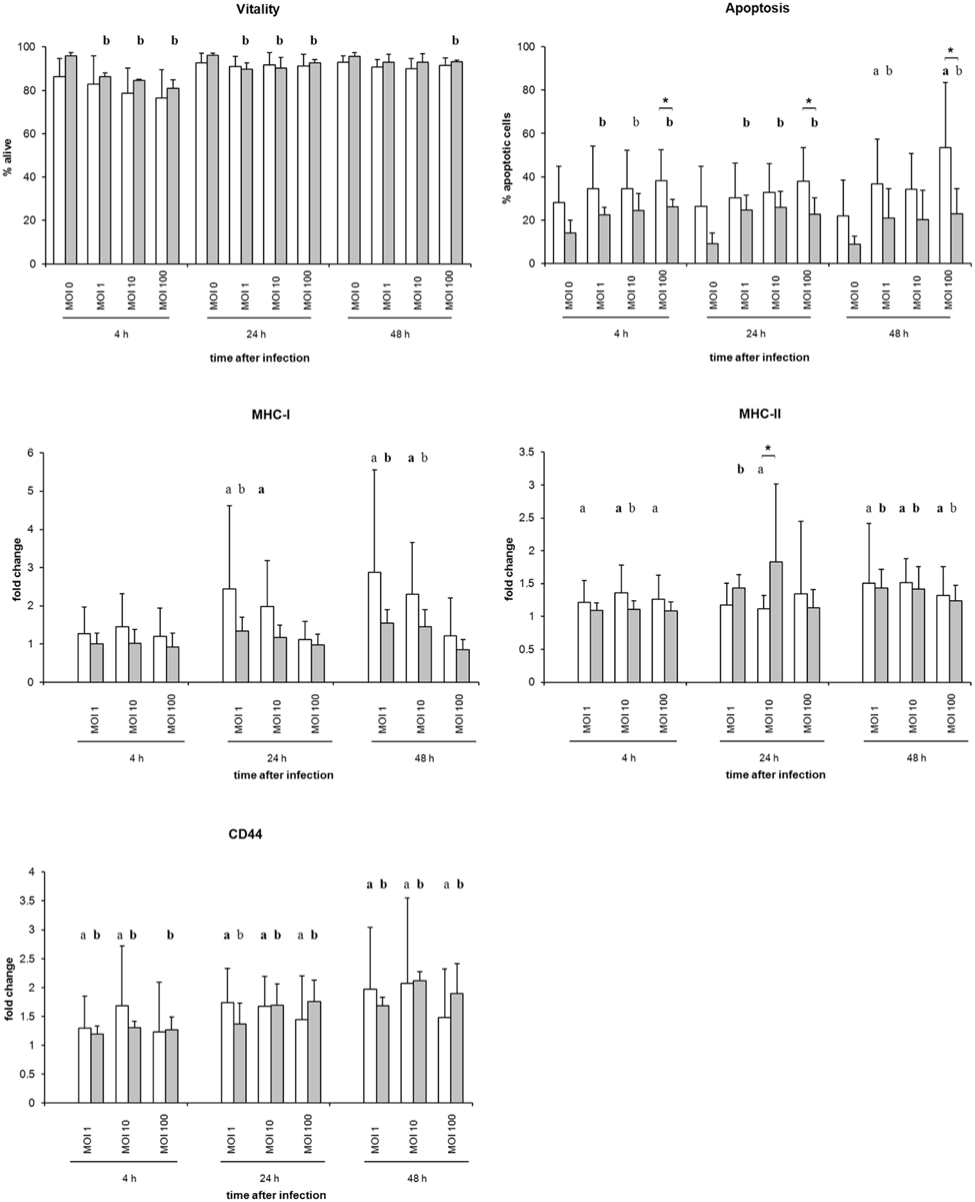
Flow cytometric analysis of primary avian macrophages after infection. The vitality of avian macrophages was defined by propidium iodide. Apoptotic cells were stained using Annexin V Fluos. Diagrams additionally show fold changes of fluorescence intensity of *S*. Typhimurium- and *S*. Infantis-infected avian macrophages after staining with different monoclonal antibodies. White columns—macrophages infected with *S*. Typhimurium (n = 4), Grey columns—macrophages infected with *S*. Infantis (n = 4) ** *P* ≤ 0.05 or * *P* ≤ 0.1 *S*. Typhimurium vs. *S*. Infantis; **a**) *P* ≤ 0.05 or a) *P* ≤ 0.1 *S*. Typhimurium vs. control; **b**) *P* ≤ 0.05 or b) *P* ≤ 0.1 *S*. Infantis vs. control.

The viability of avian macrophages was investigated by means of flow cytometry (propidium iodide staining of dead cells). After infection, avian macrophages showed more than 75% viability at 4 hpi and over 90% viability at 24 and 48 hpi (Fig. 4). There were no significant differences between the different *Salmonella* strains.

The apoptosis rate of the *Salmonella*-inoculated macrophages was calculated by use of Annexin V Fluos and flow cytometry. Untreated cells exhibited an apoptosis rate between 10 and 28%. After *Salmonella* infection, there was a slight increase (Fig. 4) with some significantly higher apoptosis rates in *S*. Typhimurium- than in *S*. Infantis-infected phagocytes (MOI 100; 4, 24 and 48 hpi; *P* ≤ 0.1).

### Macrophage antigen expression after *S*. infection

To investigate the immune response of avian macrophages after infection with *S*. Typhimurium or *S*. Infantis, the cells were analyzed by flow cytometry, and changes of expression intensity of the surface antigens MHC class I and class II, and CD44 were determined (Fig. 4).

The infection with both *Salmonella* serovars led to an increase of the macrophage antigen expression of MHC class I at 24 and 48 hpi as well as of MHC class II and CD44 at 4, 24 and 48 hpi (Fig. 4). Significant differences after the infection by *S*. Typhimurium or *S*. Infantis were not observed.

### Transcription of immune-related host genes after *S*. infection

**Fig 5:**
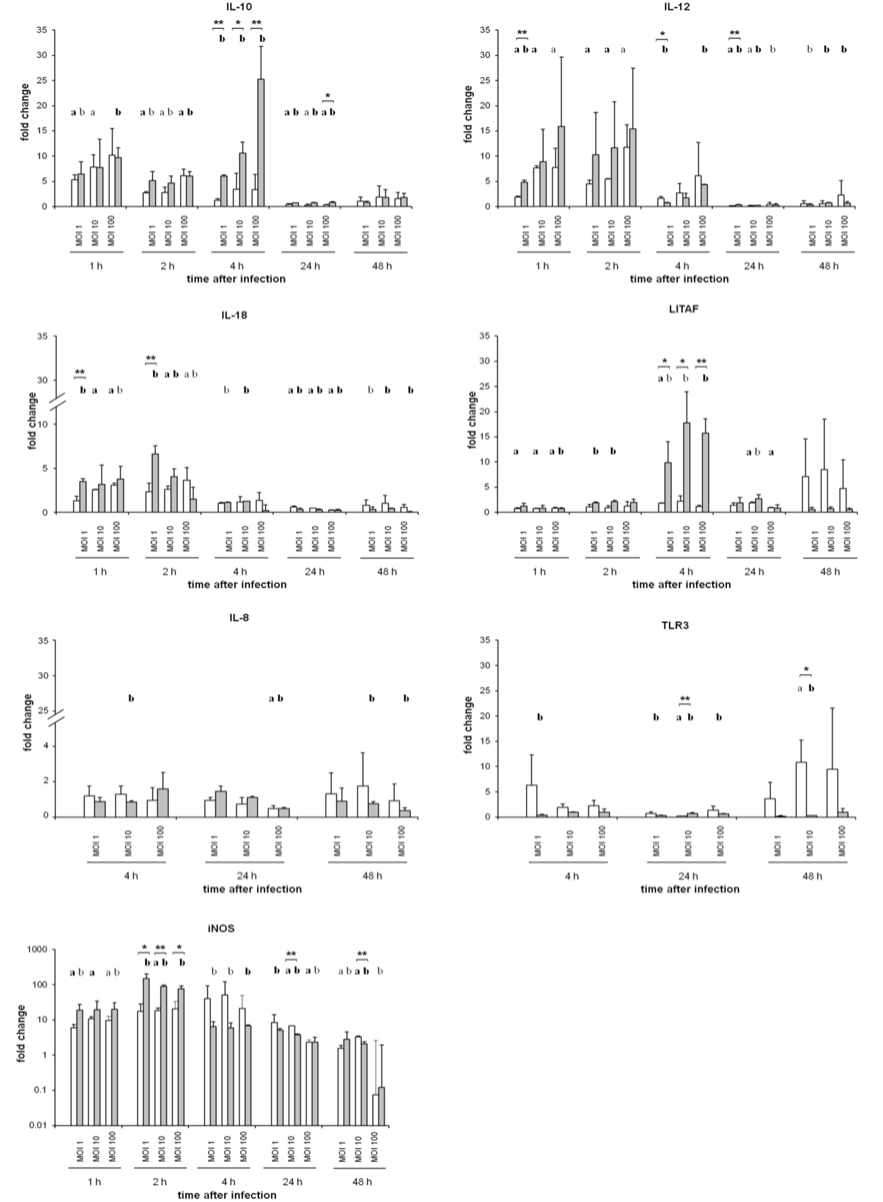
Relative quantification of mRNA expression of immune-related proteins in primary avian macrophages infected with *S*. Typhimurium or *S*. Infantis. Data were presented as fold change compared to non-infected macrophages. White columns—macrophages infected with *S*. Typhimurium (n = 6), Grey columns—macrophages infected with *S*. Infantis (n = 6) ** *P* ≤ 0.05 or * *P* ≤ 0.1 *S*. Typhimurium vs. *S*. Infantis; **a**) *P* ≤ 0.05 or a) *P* ≤ 0.1 *S*. Typhimurium vs. control; **b**) *P* ≤ 0.05 or b) *P* ≤ 0.1 *S*. Infantis vs. control.

Quantitative real-time RT-PCR revealed only sporadic *Salmonella* serovar-dependent differences of transcription levels of a selection of immune-related genes in macrophages (Fig. 5). The increased transcription of IL-10, IL-12 and IL-18 was recorded at 1 hpi in *S*. Infantis- and *S*. Typhimurium-infected macrophages and decreased from 4 hpi onwards (IL-10 at 24 hpi). *S*. Infantis-infected cells showed significantly higher (*P* ≤ 0.05) transcription rates of IL-12 (MOI 1) at 1 hpi, IL-18 (MOI 1) at 1 hpi and 2 hpi, iNOS (MOI 10) at 2 hpi, LITAF and IL-10 at 4 hpi than *S*. Typhimurium-infected macrophages. Additionally, both *S*. serovars induced a low increase of the mRNA expression of IL-8, TLR3 (Fig. 5), and TLR4 (data not shown), but without relevant differences between both infections. No increased transcription of IL-15 was detected (data not shown).

### Nitric oxide (NO) production after *Salmonella* infection

**Fig 6:**
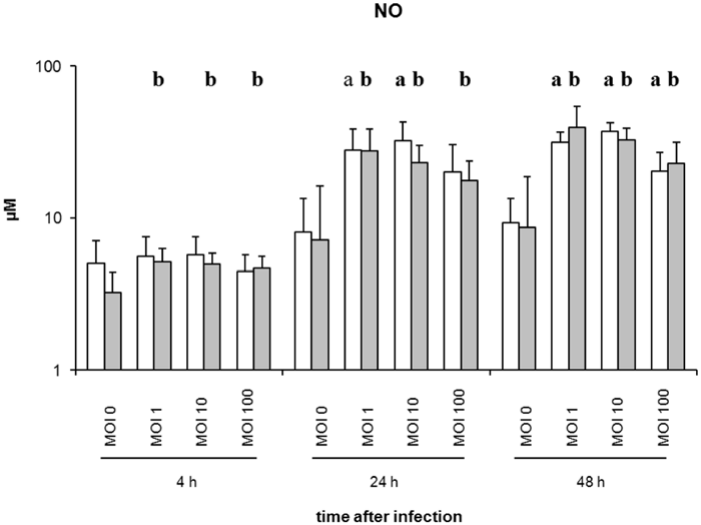
Detection of nitric oxide concentration in supernatants from avian macrophages after *Salmonella* infection. White columns—macrophages infected with *S*. Typhimurium (n = 4), Grey columns—macrophages infected with *S*. Infantis (n = 6) **a**) *P* ≤ 0.05 or a) *P* ≤ 0.1 *S*. Typhimurium vs. control; **b**) *P* ≤ 0.05 or b) *P* ≤ 0.1 *S*. Infantis vs. control.

The Griess reagent (Sigma-Aldrich) was used for spectrophotometric determination and quantification of NO in supernatant from avian primary macrophages after *Salmonella* infection (Fig. 6).

Compared to the untreated controls, experimental infection with both *Salmonella* serovars produced significantly increased NO concentrations at 24 hpi and 48 hpi (Fig. 6). At 4 hpi, significantly enhanced levels of nitrite were seen only after *S*. Infantis infection. The highest NO levels were found at 48 h after infection with *S*. Infantis (MOI 1; 39 μM/ml) and *S*. Typhimurium (MOI 10; 37 μM/ml). Using the Student’s *t* test, no significant differences between the serovars used were found.

### Transcription of *Salmonella* virulence-associated genes

**Fig 7:**
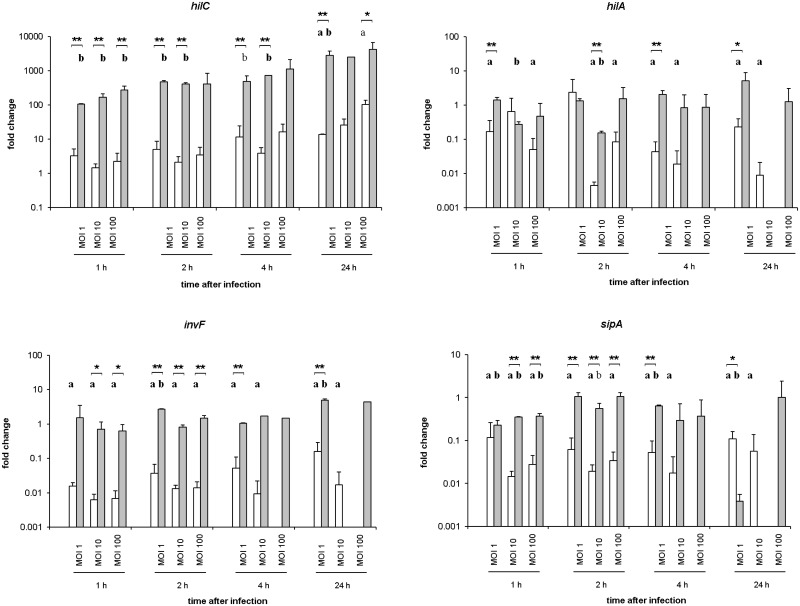
mRNA expression levels of SPI-1-associated genes of *S*. Typhimurium and *S*. Infantis in avian splenic macrophages. Data were presented as fold change compared to bacteria before infection. White columns—macrophages infected with *S*. Typhimurium (n = 6), Grey columns—macrophages infected with *S*. Infantis (n = 6) ** *P* ≤ 0.05 or * *P* ≤ 0.1 *S*. Typhimurium vs. *S*. Infantis; **a**) *P* ≤ 0.05 or a) *P* ≤ 0.1 *S*. Typhimurium vs. control; **b**) *P* ≤ 0.05 or b) *P* ≤ 0.1 *S*. Infantis vs. control.

The quantitative analysis of the transcription of ten essential *Salmonella* genes revealed significantly higher levels of the SPI-1 genes *hilC* (highest value: 2851-fold increase at 24 hpi, MOI 1; *P* ≤ 0.05) and, to a lesser extent, *hilA* (highest value: 3-fold increased at 4 hpi, MOI 1; *P* ≤ 0.05) as well as *invF* (5-fold increased at 24 hpi, MOI 1; *P* ≤ 0.05) in *S*. Infantis than in *S*. Typhimurium after infection of the macrophages (Fig. 7). The *Salmonella* gene *sipA* showed hardly any increased transcription level after infection of the cultured macrophages (Fig. 7).

**Fig 8:**
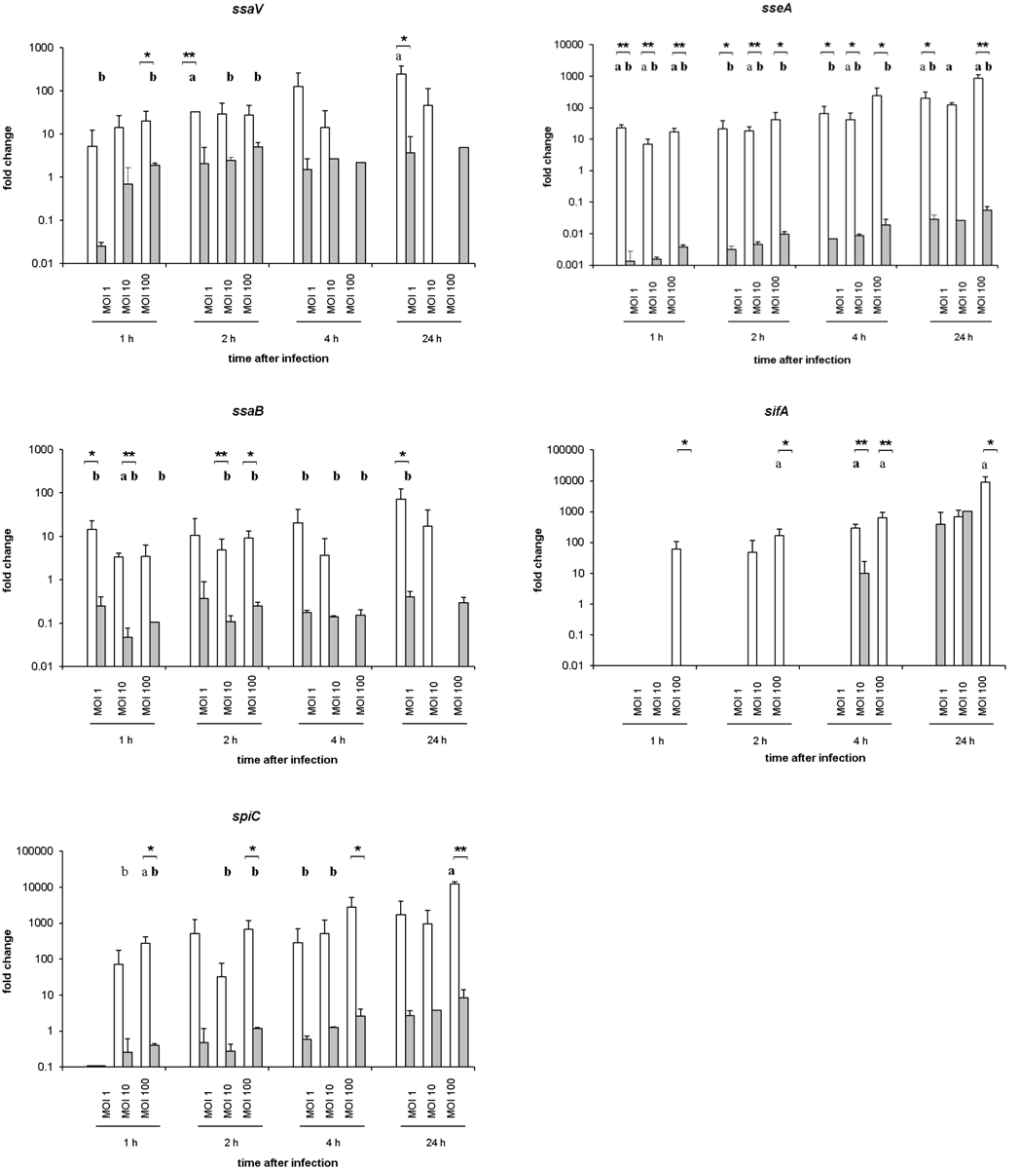
mRNA expression levels of SPI-2-associated genes of *S*. Typhimurium and *S*. Infantis in avian splenic macrophages. Data were presented as fold change compared to bacteria before infection. White columns—macrophages infected with *S*. Typhimurium (n = 6), Grey columns—macrophages infected with *S*. Infantis (n = 6) ** *P* ≤ 0.05 or * *P* ≤ 0.1 *S*. Typhimurium vs. *S*. Infantis; **a**) *P* ≤ 0.05 or a) *P* ≤ 0.1 *S*. Typhimurium vs. control; **b**) *P* ≤ 0.05 or b) *P* ≤ 0.1 *S*. Infantis vs. control.

In contrast, the mRNA expression levels of the SPI-2 genes *ssaV*, *sseA*, *ssaB*, *sifA*, and *spiC* were higher transcribed in *S*. Typhimurium than in *S*. Infantis (Fig. 8). Compared to *S*. Infantis, *S*. Typhimurium showed significantly (*P* ≤ 0.05) higher expression rates of *ssaV* (highest value: 33-fold increased at 2 hpi, MOI 1), *sseA* (highest value: 870-fold increased at 24 hpi, MOI 100), and *ssaB* (highest value: 5-fold increased at 2 hpi, MOI 10). Especially very high and significantly (*P* ≤ 0.05) increased transcription rates were found for *sifA* (highest value: 640-fold increased at 4 hpi, MOI 100) and *spiC* (highest value: 12140-fold increased at 24 hpi, MOI 100) in *S*. Typhimurium- compared to *S*. Infantis-infected cultures. In the case of *sseG*, no mRNA expression was detected (data not sown).

## Discussion

Although the mechanisms that lead to immunity against *Salmonella* are not fully understood, it is known that macrophages play a critical role in the initial recognition and control of *Salmonella* infections [2,26,27]. In the present study, an *in-vitro* infection model with primary avian macrophages was used to determine the influence of the *Salmonella* Pathogenicity Islands 1 and 2 (SPI-1 and -2) on the intracellular survivability of two non-host-specific, but *in-vivo* differently invasive, *Salmonella* serovars [6] in conjunction with the immune reaction of the host.

In the result, *Salmonella* staining and flow cytometry revealed higher percentages of macrophages containing *S*. Infantis than *S*. Typhimurium after infection. How far a possible different affinity of the used anti-LPS antibody to the serovars or different amounts of *Salmonella*-LPS per bacterium may have had an influence on these results remains unclear. The additional analysis of the SSC light intensity similarly showed differences between the serovars, but not earlier than 48 hpi. However, the flow cytometric analysis cannot differentiate between live and dead bacteria. Using microbiology detecting only living bacteria, higher numbers of intracellular *S*. Typhimurium than *S*. Infantis were found per macrophage. Thus, the results from the present study suggest that both serovars either can actively invade the cells or, perhaps, have been phagocytized by the macrophages. The observed up-regulation of the *hilC* and, to a lesser extent, the *hilA* and *invF* gene in *S*. Infantis as well as *S*. Typhimurium indicates at least the possibility of an active invasion of both bacteria strains into the phagocytes. By using immortalized avian macrophages of the HD11 cell line, other authors found that *S*. Infantis showed a greater level of invasion and/or uptake character when compared with *S*. Pullorum or *S*. Gallinarum [3]. However, in our study, *S*. Typhimurium seems to be more competent than *S*. Infantis to survive the intracellular milieu within the avian macrophages. There are no other studies having examined *S*. Infantis infection of avian primary macrophages so far. But, an *in-vitro* study with porcine alveolar macrophages showed similar results and postulated *S*. Typhimurium in comparison to *S*. Infantis as the serovar with higher virulence/survival properties [28].

For *Salmonella* a number of different strategies to ensure intracellular survival has been described [29]. Beside plasmid or bacteriophage genes, the concerted action of several bacterial factors encoded by the SPI-2 genes has been proven to be necessary to survive within host cells [30]. Indeed, in the present study, most of the SPI-2 effectors were expressed more rapidly and with higher rates in *S*. Typhimurium than in *S*. Infantis after infection of the primary avian macrophages. In contrast to our experiments, authors of a former study found the SPI-2 of *S*. Typhimurium and *S*. Enteritidis as not important for the survival of the bacteria in activated macrophage-like HD11 cells [31]. Others demonstrated the SPI-2 as important for promoting the survival of *S*. Gallinarum in HD11 macrophages [32].

For birds, the number of macrophage-specific as well as macrophage activation-related antibodies is limited. In our study, the monocyte/macrophage marker KUL01 and the anti-MHC class II marker together with morphological criteria were used to characterize the cultured splenic cells as macrophages. After *S*. Infantis and *S*. Typhimurium inoculation, an increase of the MHC class II antigen expression was found. This result, along with the increased transcription of IL-12 and iNOS, indicates an activation and polarization of the cultured cells [33,34]. In general, the avian macrophages showed *Salmonella*-triggered increased expression rates of most of the immune mediators tested, but elevated mRNA levels were solely detected up to 4 hours post infection. Former *in-vivo* studies with chicks showed a significantly augmented expression of these mediators not earlier than one or two days after infection with *S*. Infantis or *S*. Typhimurium, which persisted then up to 4 or 9 dpi [6]. The short-time reaction in our study might have been caused by LPS or SPI-1-encoded proteins of the intracellular bacteria strains. That SPI-1 genes are needed for the suppression of cytokine expression in porcine macrophages has already been described by others [35]. Beyond this, the O-group antigens have been postulated as decisive factors to influence the early innate response of chickens against *Salmonella* [36]. Whether SPI-1 encoded proteins or O-antigens can affect the cytokine expression of avian primary macrophages has to be explored in further studies.

In contrast to the short-time transcription of the cytokines, iNOS transcription as well as NO production was seen up to 48 hpi in the cell culture of our study. The production of NO is an essential factor in the host’s defense against intracellular pathogens [37]. For avian HD11 and primary macrophages, the synthesis of NO has already been described after stimulation with LPS or *Salmonella* [9,10]. However, conflicting reports on the production of NO by avian macrophage cell lines were published. He *et al*. showed a *S*. Enteritidis-induced inhibition of the NO production in HD11 cells [4], whereas Setta *et al*. demonstrated a strong induction of NO in HD11 and CKC after infection with different *Salmonella* serovars [3]. In our experiments, iNOS transcription peaked at 2 hpi for *S*. Infantis- and 4 hpi for *S*. Typhimurium-infected cells. Nevertheless, the level of NO production was similar after infection with both *Salmonella* serovars and, thus, does not explain the differences in the intracellular survivability of *S*. Infantis and *S*. Typhimurium demonstrated in this study. In further studies, it would be interesting to analyze more than one *Salmonella* strain per serovar to generalize the observed phenomena also for specific *Salmonella* O-groups.

In conclusion, both of the examined *Salmonella* serovars were able to enter the primary macrophages *in-vitro*. While the immune response of the macrophages was nearly the same for both of the bacterial strains, *S*. Typhimurium was better able to survive within these phagocytic host cells. Moreover, most of the examined SPI-2 effectors were expressed more rapidly and with higher rates in *S*. Typhimurium than in *S*. Infantis after infection of the primary avian macrophages. From this, it is very likely that *S*. Typhimurium has ensured its survival against the macrophage’s antimicrobial activity to a high degree through SPI-2 gene expression. These results strongly favor the SPI-2 and not the SPI-1 genes as the most important factors for the outcome of a *Salmonella* infection in primary avian macrophages.
